# Associations Between Human Papillomavirus Vaccine Decisions and Exposure to Vaccine Information in Social Media

**DOI:** 10.1177/10732748221138404

**Published:** 2022-11-17

**Authors:** Josheili Y. Llavona-Ortiz, Katherine E. Spanos, Jennifer L. Kraschnewski, Gail D’Souza, Jessica Gall Myrick, Kristin K. Sznajder, William A. Calo

**Affiliations:** 1 Department of Public Health Sciences, 12310 Penn State College of Medicine, Hershey, PA, USA; 2 12310 Penn State College of Medicine, Hershey, PA, USA; 3 Department of Medicine, 12310 Penn State College of Medicine, Hershey, PA, USA; 4 Donald P. Bellisario College of Communications, 311370 Pennsylvania State University, University Park, PA, USA; 5 Penn State Cancer Institute, Hershey, PA, USA

**Keywords:** human papillomavirus vaccine, vaccine refusal, social media, parents, health communication

## Abstract

**Purpose:**

Exposure to different types of vaccine information in social media can result in parents making disparate vaccine decisions, including not following national guidelines for human papillomavirus (HPV) vaccination. We sought to characterize parents’ exposure to and engagement with information about HPV vaccination in social media, and the associations between exposure to such information and vaccine decisions for their adolescent children.

**Methods:**

In 2019, we conducted a web-based survey with a national sample of 1073 parents of adolescents who use social media. The survey assessed whether parents have seen information in favor, against, or mixed about HPV vaccination. Multivariable logistic regressions assessed correlates of vaccine decisions, including HPV vaccine initiation, delay, and refusal.

**Results:**

Sixty-one percent of parents reported that their children have initiated HPV vaccination. Over one-third of parents (37%) reported seeing HPV vaccine information on social media, which was either in favor (20%), against (5%), or a mix (12%). Parents exposed to information in favor were more likely than those who saw no information to have initiated HPV vaccination (OR = 1.74, 95% CI:1.24, 2.44). Parents exposed to information against vaccination were more likely to have delayed (OR = 3.29, 95% CI:1.66, 6.51) or refused (OR = 4.72, 95% CI:2.35, 9.50) HPV vaccination. Exposure to mixed information was also significantly associated with vaccine delay and refusal.

**Discussion:**

Our findings suggest that the type of information seen on social media regarding HPV vaccination may influence the decisions parents make about vaccinating their children. Efforts should be sought to increase online information in favor of HPV vaccination and combat vaccine misinformation in social media.

## Introduction

Social media is a common source of health information for many U.S. parents,^1^ providing easy access to boundless information about numerous health topics.^2^ However, the convenient, quick sharing of information comes with risks, as social media content for some health topics like vaccines may show misinformation.^3,4^ Vaccine misinformation, including content posted on social media, contributes to vaccine hesitancy and can influence parents’ vaccine decisions.^5^ This is problematic given that parents do not have to deliberately search for vaccine information on social media to be exposed to it. For instance, parents with increased social media use are more aware of the human papillomavirus (HPV) vaccine than parents with lower or no social media use.^6^

The HPV vaccine is heavily discussed on social media,^7^ and information against HPV vaccination is both prevalent and growing.^8^ A recent study examined stories about adverse vaccination events posted on Facebook, mostly unverified, and found that the HPV vaccine was the most frequently mentioned type of vaccine in this context.^9^ Another study, summarizing 10 years of HPV vaccine-related Facebook posts, found that 45% of posts from 2006 through 2016 had messaging against vaccination (compared to 29.7% positive and 25.3% neutral) and focused on barriers more than benefits.^8^ Analyses on other social media platforms like YouTube, Twitter, and Instagram have also shown majority of the vaccine-related content to be against the HPV vaccine^10^ or a mix of information in favor and against.^11,12^ Equally concerning is the high level of engagement with the anti-vaccine content on social media, with information against HPV vaccination gaining more “likes” than posts in favor of HPV vaccination.^13^

The 2020 National Immunization Survey (NIS)-Teen reports that only 61% of adolescents 13-17 years old were up-to-date with their HPV vaccination.^14^ A growing number of studies have explored the potential role of HPV vaccine information on social media and parents’ vaccine attitudes and behaviors toward vaccinating their children.^6,15^ Dunn and colleagues found that prior exposure to tweets against HPV vaccination was associated with expressing negative opinions regarding the vaccine.^16^ Another study reported that parents who had seen information against HPV vaccination on social media were more likely to believe the vaccine to be unsafe and harmful to their child.^17^ Healthcare providers have also listed social media content against HPV vaccination as a major barrier to improve HPV vaccination rates among their adolescent patients.^18^ In this same line, Margolis et al. reported that U.S. parents who had heard stories of harm due to HPV vaccination were less likely to initiate the vaccine series and more likely to delay or refuse the vaccine compared to parents who heard no HPV vaccine stories.^19^ Interestingly, exposure to stories about diseases the HPV vaccine could have prevented (in favor of vaccination) was not associated with initiation, delay, or refusal.^19^ This prior study only assessed exposure to stories about harms and preventable diseases and focused on multiple communication channels (i.e., social media, traditional media, and conversations).^19^

Given the growing literature indicating that exposure to vaccine information posted on social media influences HPV vaccine attitudes and behaviors, it is important to understand the role of different types of vaccine information on parents’ decisions about HPV vaccination. Other studies that have evaluated such associations have used narrow definitions of information exposure (e.g., stories), focused on exposure from a single social media platform, or not assessed engagement with information after exposure. Therefore, we sought to better understand exposure to broad types of vaccine information in social media using data from a U.S. sample of parents of adolescents. The aims of our study were to (1) characterize the type of information parents had seen about the HPV vaccine on social media, (2) describe how parents engage with such information, and (3) evaluate the associations between exposure to vaccine information on social media and vaccine decisions for their adolescent children.

## Methods

### Participants and Procedures

Participants were members of an existing market research panel of U.S. adults maintained by Qualtrics, a commercial software and survey research company. The panel employs a range of recruitment methodologies to produce a sampling frame that is not overly reliant on any distinct demographic group.^20^ Invitations to our online survey were emailed to a random sample of 11 000 panel members, and 6470 responded by visiting the survey and completing the eligibility screener. Eligible participants were parents of at least one 11- to 17-year-old child living primarily in their household. We focused on U.S. parents because national guidelines approved by the Centers for Disease Control and Prevention’s (CDC) Advisory Committee on Immunization Practices (ACIP) recommend HPV vaccination for minors ages 11-17.^21^ A total of 1109 parents were eligible, provided implied consent (participants consented to answer a 10-minute online survey), and completed the survey between July and August 2019. After accounting for ineligible panel members (n = 5270) and excluding respondents who failed to complete at least two-thirds of the survey (n = 91), the survey response rate was 58%.^22^ The survey was programmed to stop recruiting when it reached 1200 participants, regardless of survey completeness. Participants with more than 1 eligible child were instructed to respond about the child with the most recent birthday. For this analysis, 36 participants who reported not using social media at the time of the survey were excluded, producing an analytic sample of 1073 parents. The Penn State College of Medicine’s Institutional Review Board approved the study protocol on September 13, 2018 (Study #00010362).

### Measures

#### HPV Vaccine Decisions (primary and secondary outcomes)

The questionnaire assessed 3 HPV vaccine decisions (i.e., initiation, delay, refusal), first orienting parents to HPV vaccination with the statement, “The next questions are about the human papillomavirus (HPV) vaccine. It’s also called Gardasil.” Our primary outcome, HPV vaccine initiation, was assessed with the question, “How many shots of the vaccine has [child’s name] had?” Response options were dichotomized into initiated HPV vaccination (“1 shot,” “2 shots,” “3 shots or more,” and “at least 1 shot, but don’t remember how many”) and not initiated (“none” and “I don’t know”). Our secondary outcomes were HPV vaccine delay and refusal. Parents were asked if they have ever delayed getting the HPV vaccine for their child with the following question (yes/no), “Has there ever been a time when you delayed or put off getting the HPV vaccine for [name]?” HPV vaccine refusal was assessed with the question (yes/no): “Has there ever been a time when you refused or decided not to get the HPV vaccine for [name]?” Vaccine delay and refusal questions were adapted from the literature and were only asked to parents who reported having discussed HPV vaccination with a healthcare provider (n = 878).^19^

#### Exposure to HPV Vaccine Information

The questionnaire assessed whether participants were exposed to information about the HPV vaccine on social media with 1 question (yes/no), “Have you seen information (e.g., stories, reports, videos, news, etc.) about the HPV vaccine on social media even when you were not looking for it?” Parents who reported seeing information were then asked to categorize it, with response options: completely in favor, mostly in favor, mostly against, completely against, or a mix of both. Based on these 2 items, we created a four-category variable of exposure that captured whether parents had seen information: in favor of HPV vaccine, against HPV vaccine, a mix (in favor and against), or neither (no reported HPV vaccine information seen on social media).

#### Characterization of and Engagement with Information Seen in Social Media

The questionnaire queried parents’ additional questions based on the reported stance of information seen. These questions were grouped in 2 blocks, in favor or against. Those who saw a mix of information (n = 126) were randomly assigned to 1 of the blocks and responded to questions either about the information they saw in favor (n = 58) or against (n = 68) HPV vaccine. We chose this process of random assignment for parents who reported seeing mixed information in order to minimize their burden taking our survey as each block of questions added eleven items. Each block of questions asked parents to further characterize the information seen, with responses including parents’ stories, news reports, information from health or medical organizations, information from advocacy groups, stories from cancer patients or survivors, celebrities’ opinions, pharmaceutical company advertisement, or something else. Parents who selected more than 1 type were prompted to choose the single information type they recalled most. Parents also indicated their engagement with the information seen on social media with the following options: read it, liked it (e.g., click on the like, heart or thumbs up button), posted content, reposted content, commented on a post, blogged or participated in an online forum, or ignored it.

#### Participant Characteristics

Sociodemographic variables of the sample included parents’ sex, age group, race/ethnicity, and educational attainment. Household characteristics were annual income and state of residence (categorized in 4 U.S. regions). The survey also assessed the sex and age of the index child.

Content and face validity were established for the survey instrument by having it reviewed by 4 researchers experienced in survey research and vaccine communication studies. More details about our measures and survey development are available elsewhere.^23^

### Data Analysis

Sociodemographic variables were reported for the full analytic sample (n = 1073) using frequencies (Table 1). Differences between parents who had seen HPV vaccine information in social media compared with those who had not seen HPV vaccine information in social media were assessed using chi-square ($\chi^{2}$) tests (data not shown). Any variable that resulted in a statistically significant difference (P < .05) was included in the multivariable logistic regression models for adjustment (i.e., parents’ age group). Models were also adjusted for demographic variables that correlate with HPV vaccine decisions, including child’s sex, child’s age group, parent’s race/ethnicity, and parent’s education.^19,24^ We reported multivariable analyses with odds ratios (OR) and their corresponding 95% confidence intervals (95% CI). We modelled type of information exposure as a categorical variable (in favor only, against only, mixed, and neither [reference group]). We conducted separate regression analyses for each HPV vaccination decision (i.e., initiation, delay, and refusal). The first model (initiation) compared parents who had versus had not initiated the HPV vaccine series for their children using the full sample. The other 2 models (delay and refusal) compared parents who reported delaying or refusing HPV vaccination versus those that had not within a subset of parents who reported discussing HPV vaccination with their child’s provider. Analyses were conducted using STATA/BE^25^ and all statistical tests were performed at the .05 significance level.

**Table 1.** Characteristics of a national sample of parents of 11- to 17-year-old adolescents, 2019 (n = 1073).

	n (%)
Child characteristics	
Sex	
Male	526 (49)
Female	547 (51)
Age group	
11-12 years old	320 (30)
13-17 years old	753 (70)
Parent characteristics	
Sex	
Male	264 (25)
Female	809 (75)
Age group	
18-34 years old	184 (17)
35-45 years old	540 (50)
46 years or older	349 (33)
Race/Ethnicity	
Non-Hispanic White	764 (71)
Non-Hispanic Black	130 (12)
Hispanic	121 (11)
Other	58 (6)
Education	
High school or less	243 (23)
Some college	444 (41)
College degree or higher	386 (36)
Household characteristics	
Annual Income	
$39,999 or less	327 (30)
$40K to $79,999	348 (32)
$80K or higher	363 (34)
Not reported	35 (4)
Region	
Northeast	147 (14)
Midwest	214 (20)
South	327 (30)
West	385 (36)

## Results

### Participant Characteristics

Parents were split between reporting on a daughter (51%) or a son (49%) (Table 1), and the mean age of the reported child was 14 years (SD: 1.9 years). Three-quarters of parents were female (75%) and a majority were non-Hispanic white (71%), with good representation of non-Hispanic blacks (12%) and Hispanics (11%). Almost one-fourth (23%) of parents had a high school degree or less, and almost one-third (30%) reported an annual household income of less than $40,000. Over one-third of parents (n = 395; 37%) reported seeing HPV vaccine information in social media. Parents who reported exposure to HPV vaccine information were younger than those who have not seen such information in social media (P < .05).

### HPV Vaccine Information Seen on Social Media

Overall, 20% of parents reported seeing information in favor of HPV vaccination, 5% against HPV vaccination, and 12% saw a mix of information in favor and against HPV vaccination. Among parents who reported seeing favorable information (n = 275), the most recalled content was from health or medical organizations (30%), followed by parents’ stories (20%), pharmaceutical or industry advertisement (16%), and news reports (16%) (Table 2). On the other hand, among parents who reported seeing information against HPV vaccine (n = 120), the most recalled information was parents’ stories (51%), followed by news reports (18%), and information from advocacy groups (12%).

**Table 2.** Most recalled HPV vaccine information seen on social media among parents of adolescents (n = 395).

	In favor (n = 275) n (%)	Against (n = 120) n (%)
Parents’ stories	56 (20)	61 (51)
News reports	43 (16)	21 (18)
Information from health or medical organizations	82 (30)	10 (8)
Information from advocacy groups	19 (7)	14 (12)
Stories from cancer patients or survivors	15 (5)	2 (2)
Pharmaceutical or industry advertisement	44 (16)	3 (2)
Something else	16 (6)	9 (7)

### Engagement With HPV Vaccine Information

Parents who had seen information in favor of HPV vaccination reported varying levels of engagement with the content, including reading (76%), liking (23%), posting (10%), and commenting (9%) (Table 3). Parents who had seen information against HPV vaccine also reported high levels of engagement, in terms of reading the content (68%). Parents exposed to negative vaccine information also reported commenting (15%), liking (12%), and reposting the content (10%). One-fifth (20%) of parents who saw information in favor of HPV vaccination ignored the content compared with almost one-third (30%) of those who saw information against the vaccine.

**Table 3.** Parent’s engagement with HPV vaccine information seen on social media (n = 395).

	In favor (n = 275) n (%)*	Against (n = 120) n (%)^a^
Read it	210 (76)	81 (68)
Liked a post	64 (23)	14 (12)
Posted content	28 (10)	8 (3)
Reposted content	22 (8)	12 (10)
Commented on a post	25 (9)	18 (15)
Blogged or participated in an online forum	14 (5)	2 (2)
Ignored it	54 (20)	36 (30)

^a^Does not sum up to 100% because participants could select more than 1 option.

### Associations Between Exposure to Information and HPV Vaccine Initiation

Sixty-one percent (n = 659) of children had initiated HPV vaccination at the time of this survey. Out of 217 parents that indicated seeing only information in favor of HPV vaccine on social media, over two-thirds (n = 156; 72%) reported that they had initiated HPV vaccination. In a multivariable analysis, parents who only saw information in favor of HPV vaccination were more likely (OR = 1.74, 95% CI: 1.24, 2.44) to have initiated HPV vaccination than those who had seen no information (Table 4). Exposure to either information against HPV vaccination only or mixed was not associated with initiating the HPV vaccine series.

**Table 4.** Associations between HPV vaccine decisions and exposure to vaccine information in social media, findings from a sample of U.S. parents of adolescents.

Category of information exposure	Parents reporting HPV vaccine initiation/Total parents per category^a^			Parents reporting HPV vaccine delay/Total parents per category^b^			Parents reporting HPV vaccine refusal/Total parents per category^b^		
	n/N (%)	OR	(95% CI)	n/N (%)	OR	(95% CI)	n/N (%)	OR	(95% CI)
In favor	156/217 (72)	1.74	(1.24, 2.44)	41/181 (23)	1.19	(.79, 1.80)	31/181 (17)	1.13	(.71, 1.79)
Against	27/52 (52)	.70	(.39, 1.25)	17/38 (45)	3.29	(1.66, 6.51)	17/38 (45)	4.72	(2.35, 9.50)
Mixed	74/126 (59)	1.00	(.67, 1.47)	39/102 (38)	2.47	(1.56, 3.90)	25/102 (25)	1.70	(1.01, 2.86)
Neither	402/678 (59)	Ref		99/496 (20)	Ref		80/496 (16)	Ref	

Note: All models adjusted for child’s sex, child’s age group, parent’s race/ethnicity, parent’s education, and parent’s age group.

OR = odds ratio; CI = confidence interval; Neither = Parents who have not seen HPV vaccine information in social media.

^a^Model compared parents who had vs had not initiated HPV vaccination, using the full sample (n = 1073).

^b^Model compared parents who reported delaying or refusing HPV vaccination vs those that had not, in the subset of parents who reported discussing vaccination with their child’s provider (n = 878).

### Associations Between Exposure to Information and HPV Vaccination Delay and Refusal

Among 878 parents who reported having discussed HPV vaccination with their child’s provider, almost one-fourth (n = 196; 22%) said that they have delayed their child getting the vaccine, and 17% (n = 153) reported refusing HPV vaccination for their child. Parents who had seen information against HPV vaccination only were more likely to have delayed (OR = 3.29, 95% CI: 1.66, 6.51; Table 4) the vaccine series when compared to parents who reported no information exposure. Those who had seen mixed information were also more likely (OR: 2.47; 95% CI: 1.56-3.90) to have delayed HPV vaccination. Likewise, parents who had been exposed to information against HPV vaccination only (OR = 4.72; 95% CI: 2.35, 9.50) or mixed information (OR = 1.70; 95% CI: 1.01, 2.86) were more likely to have refused HPV vaccination when compared to parents who were not exposed to HPV vaccination information. Exposure to information in favor of HPV vaccination only was not associated with either delay or refusal.

## Discussion

In this study using a national sample of U.S. parents of adolescents, we found that parents who had seen information against HPV vaccination in social media, whether alone or mixed with information in favor, more often decided to delay or refuse vaccination when compared to those who did not see any information. These findings are consistent with the literature showing that parents who had heard stories about HPV vaccine harms are more likely to delay or refuse the vaccine series for their children.^19^ Similarly, recent communication experiments show that participants exposed to a negative blog about HPV vaccination^26^ or tweets with misinformation had reduced intentions to vaccinate.^27^ On the other hand, parents exposed to information in favor of vaccination were more likely than those who saw no information to have initiated HPV vaccination. These findings deviate from those reported by Margolis and colleagues who showed no association between hearing positive stories about HPV vaccination and vaccine initiation.^19^ The prior study asked participants about stories they had heard specifically on HPV vaccine preventable diseases through multiple sources, including social media, traditional media, and conversations with people.^19^ In contrast, our study asked about HPV vaccine information more broadly and only on social media. Despite these methodological differences, the studies align in suggesting that negative vaccine content in social media can be a deterrent to HPV vaccination, even when mixed with positive messaging.

Overall, 37% of parents in our sample reported seeing information about the HPV vaccine on social media, similar to a prior report showing 41% of information exposure among U.S. parents of adolescents who had not yet completed the HPV vaccines series.^19^ Other authors have reported as high as 58% of parents being exposed to HPV vaccine information in social media, but the data were collected using convenience samples.^17^ About one-third (32%) of parents in our study had seen favorable vaccine information, which is almost twice as many who reported seeing information against vaccination (17%), whether alone or mixed. Our finding aligns with recent studies showing that social media content supporting HPV vaccination is more prevalent and reaches more users than negative messaging. For example, Massey et al. reported that 39% of tweets about HPV vaccination express positive sentiment versus 25% of tweets that showed negative sentiment, and that a larger number of Twitter users were exposed to positive sentiment than to negative sentiment.^28^ However, despite having a more limited level of exposure, anti-vaccine information posted on social media is especially powerful in influencing parents to decide against on-time HPV vaccination for their children, as shown by our results and those of other studies.^15,16,19^ This aligns with research showing that negative information is more influential in shaping perceptions and decisions than positive information.^29^ Public health messaging demonstrating the benefits of HPV vaccination might consider first showing the potential health risks (e.g., developing vaccine-preventable cancers) when one does not vaccinate. Also, public health campaigns promoting HPV vaccination on social media should be more frequent so that the negative content is less likely to be seen and, as such, becomes slightly less influential.

This study also found interesting results about the characteristics of information seen by parents. Among parents who reported seeing information in favor of HPV vaccine, almost one-third (30%) said that the most recalled content was from health or medical organizations. Recent content analyses of social media show that health-related users or advocates (e.g., public health, medical, and research organizations) create significantly more positive HPV vaccine content than other users.^12,13^ Parents’ stories were the second most recalled information in favor of vaccination (20%), but more than half (51%) of parents who reported seeing information against vaccination recalled these stories from other parents the most. Kearny et al. similarly reported that personal stories about HPV vaccine on Instagram were significantly more anti-vaccine (55%) than pro-vaccine (45%).^13^ Because of the potential persuasiveness of these personal stories, many healthcare organizations and vaccine advocates are now promoting the use of stories from parents and cancer survivors to reach and educate the general public about the importance of HPV vaccination, especially for individuals with low literacy.^27^ Moreover, a recent study with U.S. parents of children ages 9-14 indicated that parents want to learn about the HPV vaccine through other parent experiences, especially when the information aligns with science supporting the vaccine.^32^ We found that news reports seen on social media were a common source of information against vaccination. Keim-Malpass et al. reported that news and lay media were a common source of tweets regarding HPV vaccination with almost one-fourth (24%) showing negative sentiment and the rest being positive or neutral.^12^ As such, training for journalists or health-related communicators may be needed to help counter misleading information about vaccination that appears from news outlets on social media.

A majority of parents who saw information, either in favor or against vaccination, reported engaging with that content, mostly reading it, but also by liking, commenting, or reposting it. Other studies have reported mixed findings in terms of engagement. For example, pro-vaccine posts were liked significantly less frequently than anti-vaccine posts in a study evaluating HPV vaccine content on Instagram.^13^ Another study reported that positive-sentiment tweets about HPV vaccination generate a significantly higher number of retweets per tweet than negative tweets.^28^ Studies have also shown that engagement with social media content varies depending on features like showing images, hashtags, tags, or links, as those features may provide higher visual appeal and content credibility.^30^ Our study did not ask participants about the prevalence of these features on the social media content they saw but the literature shows that these are common.^28^ We also know from prior research that the type of information is important for engagement. For example, Kearny et al. reported that personal stories receive nearly twice as many likes as informational posts about HPV vaccination in Instagram.^13^ Furthermore, attempts to reach parents using personal stories on Facebook have generated high engagement and positive dialogue around the HPV vaccine,^31^ suggesting that social media could be a promising space to disseminate HPV vaccine information if it is done in a credible way.^32^ Future studies should evaluate what strategies can be used to increase the credibility of trusted sources of vaccine information (e.g., CDC’s or medical associations’ social media accounts) and the role that literacy plays in a person trusting in one social media source more than another.

A previous study by our team found that many parents also support measures that would aid in social media becoming a space with accurate vaccine information. We found that 61% of U.S. parents of adolescents were in favor of implementing at least 1 social media standard to reduce vaccine misinformation.^23^ The most supported standard by parents was to fact-check the information before it is posted online (51%), which allows social media platforms to flag it as inappropriate beforehand.^23^ Moreso, the study found that parents who had been exposed to information in favor of the HPV vaccine were more likely to support social media standards to combat vaccine misinformation, again showing the potential positive impact of favorable vaccine information on social media as in this study.^23^ Other studies also report that social media messaging about HPV vaccination, including personal stories, from healthcare professionals can help engage parents in vaccine conversations and motivate them to vaccinate their children.^31,32^ With our finding that the most recalled content by parents who had seen favorable HPV vaccine information came from health or medical organizations (30%), it is important to seek approaches to motivate healthcare professionals to continue disseminating science-based information about vaccines on social media. Equally important, when communicating with parents during child wellness visits, providers should use presumptive announcements to clearly recommend HPV vaccination and address parents’ concerns with evidence-based messages.^33^ Providers can use this opportunity to understand whether parents’ vaccine concerns stem from misinformation they have seen on social media and then, provide answers appropriate to parents’ literacy level or direct them to trusted resources where they can access accurate vaccine information.

### Strengths and Limitations

Study strengths include using a large, national sample of parents and an adequate response rate. Importantly, the proportion of minority participants in the study approximates the national distribution of non-Hispanic black and Hispanic adult groups. This study had several limitations. Our questionnaire only asked parents about information specific to the HPV vaccine that they have seen on social media. It is possible that parents saw information either in favor or against vaccines from other modes of information delivery that may have contributed to their attitudes and decisions regarding HPV vaccination. Temporality of events was not assessed and, therefore, it is unknown if the information parents reported exposure to on social media was seen before or after their child’s vaccination decision or maybe on a more continuous basis. Moreover, our study did not assess the level of engagement with each social media platform that respondents reported using, limiting our ability to understand varying exposure to HPV vaccine information across platforms. Lastly, this study relied on self-reported data for our vaccine decision variables without verifying participant responses using immunization records, but studies show that parents’ recall of this information is fairly accurate.^34^

## Conclusion

Our study suggests that the type of information seen on social media regarding HPV vaccination may play a role in the decisions parents make about vaccinating their children. Efforts should be sought to increase online information in favor of HPV vaccination and combat vaccine misinformation on social media. Future approaches should seek to strengthen the inclusion of healthcare professionals in interventions to provide factual vaccine information considering that healthcare organizations represented the most recalled content of parents who had seen information in favor of the HPV vaccine.

### List of Abbreviations/Acronyms

CI-Confidence Interval, HPV-Human Papillomavirus, NIS-National Immunization Survey, OR-Odds Ratio, SD-Standard Deviation, CDC-Centers for Disease Control and Prevention, ACIP-Advisory Committee on Immunization Practices.
